# Antibacterial activity of baicalein against *Aeromonas hydrophila*: *in vitro* and *in vivo* evaluation

**DOI:** 10.3389/fmicb.2025.1615029

**Published:** 2025-07-14

**Authors:** Reng Qiu, Jun Li, Changjun Jiang, Yang Yu, Dandan Li, Xuehan Xie, Yang Lei, Lunguang Yao

**Affiliations:** ^1^Henan Key Laboratory of Insect Biology in Funiu Mountain, Henan Field Observation and Research Station of Headwork Wetland Ecosystem of the Central Route of South-to-North Water Diversion Project, College of Water Resources and Modern Agriculture, Nanyang Normal University, Nanyang, China; ^2^School of Biological Sciences, Lake Superior State University, Sault Ste. Marie, MI, United States; ^3^Henan Key Laboratory of Insect Biology in Funiu Mountain, College of Life Science, Nanyang Normal University, Nanyang, China; ^4^Aquatic Life Epidemic Prevention Center of Huidong, Agriculture and Rural Bureau of Huidong, Huizhou, China; ^5^Henan Key Laboratory of Insect Biology in Funiu Mountain, Collaborative Innovation Center of Water Security for Water Source Region of Mid-route Project of South-North Water Diversion of Henan Province, Nanyang Normal University, Nanyang, Henan, China

**Keywords:** baicalein, *Aeromonas hydrophila*, antibacterial, biofilm, grass carp

## Abstract

**Introduction:**

*Aeromonas hydrophila* (AH) is a pathogenic bacterium commonly found in aquatic organisms, particularly in fish products. Baicalein, a bioactive flavonoid derived from traditional Chinese herbal medicine, possesses a wide range of pharmacological properties, including anticancer, antibacterial, antiviral, antioxidant, hepatoprotective, and anti-inflammatory effects.

**Methods:**

*In vitro*, the Oxford cup method was employed to assess the antibacterial activity of baicalein, while the minimum inhibitory concentration (MIC) and minimum bactericidal concentration (MBC) were determined using the microtiter broth dilution technique. Biofilm formation and bacterial motility were evaluated through the crystal violet assay and swimming assay, respectively. The impact of baicalein on bacterial membrane permeability and morphology was observed using the Live/Dead BacLight kit and transmission electron microscopy. *In vivo*, grass carp were used as the model organism to evaluate the effect of baicalein on AH proliferation, while the expression of pro-inflammatory cytokines and antioxidant factors was quantified by qRT-PCR.

**Results:**

This study demonstrated the antibacterial activity of baicalein against AH infection. Baicalein exhibited bacteriostatic effects *in vitro*, with an MIC of 40 μg/mL and an MBC of 80 μg/mL. Time-kill assays confirmed its bactericidal properties. Additionally, baicalein inhibited biofilm formation and reduced bacterial motility. The antibacterial mechanism of baicalein involved increased membrane permeability and structural disruption of AH cells. *In vivo* studies in grass carp revealed a dose-dependent reduction in AH burden following baicalein administration. Moreover, baicalein suppressed the expression of pro-inflammatory cytokines, including interleukin-1β (IL-1β), interleukin-8 (IL-8), and tumor necrosis factor-α (TNF-α), while enhancing the expression of antioxidant-related genes, such as catalase (CAT), glutathione reductase (GR), and superoxide dismutase (SOD). These findings indicate that the antibacterial and anti-inflammatory effects of baicalein contribute to its protective role against AH infection *in vivo*.

**Discussion:**

Baicalein effectively inhibits the proliferation of AH both *in vitro* and *in vivo*, highlighting its potential as a promising pharmacotherapeutic agent for the prevention of AH infections in fish.

## Introduction

*Aeromonas hydrophila* (AH) is a Gram-negative bacterium belonging to the Aeromonadaceae family, commonly found in natural water bodies and recognized as a primary pathogen of various aquatic animals. AH infections have significantly hindered the growth of China's aquaculture industry (Semwal et al., 2023). Traditional strategies for disease prevention and treatment in aquaculture primarily rely on medications and vaccinations. However, the high cost, limited efficacy, and narrow applicability of aquatic vaccines, combined with the rapid mutation of pathogens, have restricted their widespread use. Consequently, drug therapy remains the predominant approach for disease control in aquaculture. Antibiotics are widely used as the primary treatment in aquaculture. However, overuse has contributed to the emergence of antibiotic-resistant bacteria and the horizontal transfer of resistance genes to humans. Additionally, accumulation of antibiotic residuals in aquatic products has also posed significant threats to public health and the environment (Zheng et al., 2021; Zainab et al., 2020). In response, many countries have introduced regulations aimed at reducing or eliminating antibiotic resistance in aquaculture, emphasizing the need for alternative, non-resistant antimicrobial agents (Lulijwa et al., 2020).

Herbal remedies, rich in natural antibacterial compounds, offer promising alternatives to conventional antibiotics due to their efficacy and safety (Vaou et al., 2021; Woo et al., 2023). Various medicinal herbs used in aquaculture have demonstrated antibacterial properties (Zhang et al., 2022). These herbs contain bioactive compounds such as polysaccharides, essential oils, alkaloids, saponins, polyphenols, flavonoids, anthraquinones, and terpenoids, which effectively prevent and treat bacterial infections caused by *Aeromonas* spp., *Streptococcus* spp., *Vibrio* spp., and other pathogens (Zhu, 2020).

**Figure 1:**
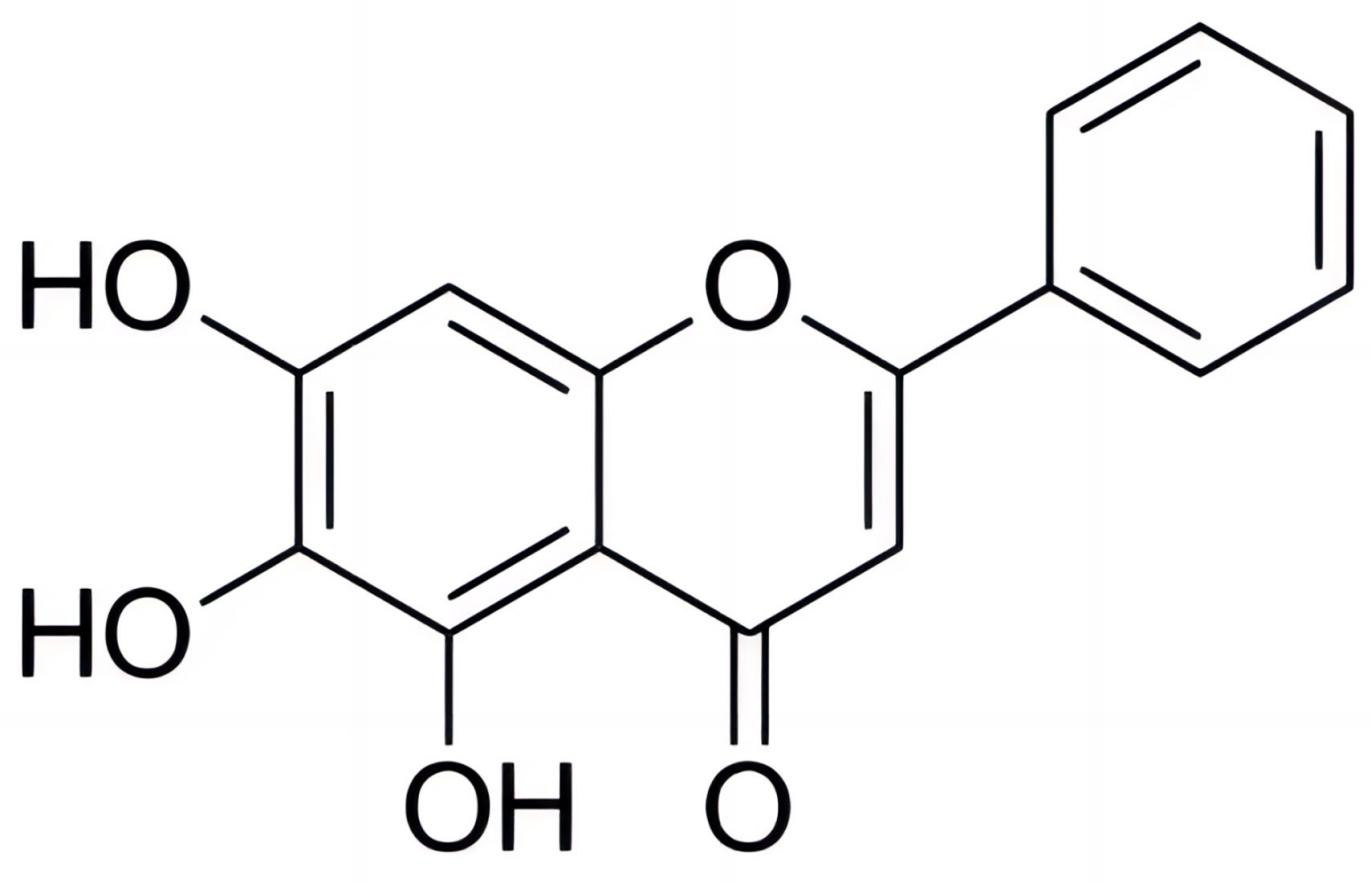
Chemical structure of baicalein. The IUPAC name of baicalein is 5,6,7-Trihydroxy-2-phenylchromen-4-one, or also known as 5,6,7-trihydroxyflavone.

Baicalein, a flavonoid (Figure 1), is a major active component of *Scutellaria baicalensis* (commonly known as Baikal skullcap) (Semwal et al., 2023). It has been well known for its low toxicity and diverse pharmacological effects, including anticancer, antibacterial, antiviral, hepatoprotective, anti-inflammatory, hypoglycemic, antithrombotic, neurogenic, cardioprotective, and wound-healing properties (Paul et al., 2024). Baicalein has demonstrated broad-spectrum antibacterial activity against both Gram-positive and Gram-negative bacteria, including ESKAPE pathogens (*Enterococcus faecium, Staphylococcus aureus, Klebsiella pneumoniae, Acinetobacter baumannii, Pseudomonas aeruginosa*, and *Enterobacter* spp.) (Morimoto et al., 2023), as well as cariogenic, periodontopathogenic bacteria (Jang et al., 2014), and *Helicobacter pylori* (Chen et al., 2018). As for the aquaculture disease aspects, the extract from aerial part of *Scutellaria baicalensis* can inhibite the growth of several common pathogenic bacteria in aquaculture that including *Aeromonas hydrophila, Edwardsiella tarda, Vibrio alginolyticus* and *Vibrio harveyi* (Xia et al., 2020). It can also serve as a replacement for antibiotics in fighting against pathogenic bacteria (Xia et al., 2023). Baicelein can improves growth performance, antioxidant activity, and intestinal flora of koi carp (*Cyprinus carpio*) (Du et al., 2022). However, no prior study has evaluated baicalein's efficacy against AH.

In this study, we investigated the antibacterial effects of baicalein on AH, focusing on its ability to inhibit bacterial growth and biofilm formation *in vitro*. Additionally, we explored its *in vivo* antibacterial, anti-inflammatory, and antioxidant effects in grass carp (*Ctenopharyngodon idella*). The findings suggest that baicalein holds potential as a plant-derived therapeutic agent for controlling AH infections in aquaculture species.

## Materials and methods

### Fish and bacterial preparation

Grass carp (*Ctenopharyngodon idella*) (81.6 g ± 8.9 g) were obtained from a local fish farm in Nanyang, Henan, China. The fish were acclimated in aerated freshwater at 23 ± 1°C for 2 weeks prior to experimentation. To confirm the absence of bacterial infections, blood, liver, and kidney samples were examined before the experiments according to previous report (Qiu et al., 2021). The fish were euthanized using 200mg/L MS-222 (Sigma, St. Louis, MO, USA) before the sacrifice or infection experiments (Qiu et al., 2021) and approved by the institutional Animal Care and Use Committee (Protocol NYNU-2022-016) to ensure animal welfare. The bacterial pathogen *A. hydrophila* (AH) was isolated from grass carp and cultivated at Nanyang Normal University, China. AH was cultured in Luria-Bertani (LB) broth at 28°C.

### Antibacterial activity

The antibacterial activity of baicalein (Aladdin, Shanghai, China) against AH was evaluated using the Oxford cup diffusion method. Baicalein was dissolved in dimethyl sulfoxide (DMSO) (Kemiou, Tianjin, China) at concentrations of 5 mg/mL and 10 mg/mL. 10 mg/mL kanamycin was used as a positive control. AH was adjusted to 1 × 10^7^ colony-forming units (CFU)/mL in phosphate-buffered saline (PBS) and spread onto LB agar plates. Sterile Oxford cups were used to create wells in the agar, into which 50 μL of baicalein solution was added. The plates were incubated at 28°C for 16–20 h, and antibacterial activity was assessed by measuring the diameter of the inhibition zones.

The minimum inhibitory concentration (MIC) was determined using the broth microdilution method. Baicalein concentrations ranging from 320 μg/mL to 1.25 μg/mL were prepared with 198 μL of AH suspension and 2 μL of the twofold serial dilutions of baicalein in 96-well plates. Wells were inoculated with AH at 1 × 10^6^ CFU/mL and incubated at 28°C for 24 h. The control group was set up by adding the same volume of DMSO and LB medium. Three parallel experiments were conducted for each group. MIC was defined as the lowest concentration at which no turbidity was observed. The minimum bactericidal concentration (MBC) was determined by plating 100 μL from clear wells onto LB agar and incubating at 28°C for 24 h. MBC was defined as the lowest baicalein concentration that killed 99.9% of the bacterial population, following CLSI M07-A8 guidelines. All assays were conducted in triplicate in two independent experiments.

### Time-kill assay

Time-kill kinetics of baicalein against AH were evaluated by culturing AH in LB broth at 1 × 10^6^ CFU/mL and treating it with baicalein at concentrations of 10, 20, 40, and 80 μg/mL. Cultures were incubated at 28°C, and bacterial viability was assessed at 0, 2, 4, 6, 8, 12, and 24 h post-treatment by determining CFU/mL. Experiments were conducted in triplicate.

### Biofilm formation assay

The effect of baicalein on biofilm formation was assessed using a crystal violet assay (Solarbio, Beijing, China) in a 96-well plate as previously described (Bhattacharyya et al., 2017). AH (2 × 10^5^ CFU/mL) was incubated in LB broth with baicalein at 2.5, 5, 10, 20, and 40 μg/mL for 72 h at 28°C. Biofilms were fixed with 100% methanol for 15 min, air-dried, and stained with 0.1% crystal violet for 20 min. After washing, the bound dye was solubilized with 30% glacial acetic acid, and biofilm formation was quantified by measuring optical density at 570 nm (OD_570_). Biofilm formation was normalized using OD_600_ (bacterial concentration), calculated as: biofilm OD_570_ (relative value) = OD_570_ (measured value)/OD_600_ (bacterial concentration).

### Motility assay

Bacterial motility was assessed using a modified method (Husain et al., 2017). Soft LB agar (0.3%) was autoclaved at 121°C for 30 min. After cooling to 55°C, baicalein was added to achieve final concentrations of 2.5, 5, 10, 20, and 40 μg/mL before plating. AH cultures were washed with PBS, adjusted to 1 × 10^7^ CFU/mL, and inoculated (2 μL) onto the agar surface. Plates were incubated at 28°C for 24 h, and colony diameters were measured. Each assay was performed in triplicate.

### Membrane permeability assay

A Live/Dead BacLight kit (Solarbio, Beijing, China) was used to assess membrane permeability. Log-phase AH cultures were treated with baicalein (2 × MIC) for 2 h, washed with 0.85% NaCl, and centrifuged at 3,000 × *g* for 5 min. Control samples were incubated without baicalein. Bacterial suspensions (1 × 10^8^ CFU/mL) were stained with SYTO 9 and propidium iodide (PI) and incubated in the dark for 15 min. Fluorescence images were captured using a Zeiss Axio Observer 7 microscope (Zeiss, Jena, Germany).

### Transmission electron microscopy

The structural effects of baicalein on AH were investigated by a Hitachi HT7700 Transmission Electron Microscope (Hitachi, Tokyo, Japan). Briefly, AH cultures were treated with baicalein (2 × MIC) for 6 h at 28°C, washed with PBS, and centrifuged at 3,000 × g for 5 min. Cells were fixed in 2.5% glutaraldehyde at 4°C overnight, washed, and air-dried before TEM analysis. DMSO- treated bacteria served as controls.

### Bacterial burden assay

Grass carp were intraperitoneally injected with 0.1 mL of AH (1 × 10^6^ CFU/mL). After 2 h, fish were randomly assigned to five groups (*n* = 5) and administered baicalein (10 mg/kg, 5 mg/kg, or 2.5 mg/kg), DMSO, or PBS via intraperitoneal injection. Fish were euthanized after 4 h, and liver and spleen samples were collected, weighed, and homogenized in PBS. Serial dilutions were plated on LB agar and incubated at 28°C for 24 h. Bacterial counts were expressed as CFU/g of tissue.

### RNA extraction and qRT-PCR

The expression of inflammation-related genes (Table 1), including interleukin−1β (*IL-1*β), interleukin−8 (*IL-8*), and tumor necrosis factor-α (*TNF-*α) and antioxidant-related genes catalase (*CAT*), glutathion reductases (*GR*) and superoxide dismutase (*SOD*) in liver tissue was analyzed via qRT-PCR. Total RNA was extracted using the Trizol method and reverse-transcribed into cDNA with Superscript II reverse transcriptase (Takara, Dalian, China). qRT-PCR was performed using a SYBR Green Pro Taq qPCR Kit (Agbio, Changsha, China) with β*-actin* as the internal control. The PCR reaction system comprised 10 μL, with 5 μL of SYBR, 0.5 μL of each primer, 3.5 μL of DEPC water, and 1 μL of cDNA as the template. The PCR conditions were: 95°C for 5 min, and followed by 40 cycles of 95°C for 10 s, 60°C for 15 s, and 72°C for 15 s. The relative gene expression levels were calculated using the 2^−ΔΔCt^ method.

**Table 1:** Primers used in this study.

**Primer name**	**Primer sequence (5^′^-3^′^)**	**GenBank accession**
qIL-1β-F	CTGGTCTTGGAGGAGGTCACTGAA	JN705663.2
qIL-1β-R	CTACTTGGCACCTGGCACACTTC	
qIL-8-F	GCTGTGGCATGTCTGACCATTACT	JN255694.1
qIL-8-R	ACAGTGAGGGCTAGGAGGGTAGA	
qTNF-α-F	TGCTGTCTGCTTCACGCTCAAC	JQ670915.1
qTNF-α-R	AGCCTGGTCCTGGTTCACTCTC	
qCAT-F	TGCCTGTCAACTGCCCCTAC	FJ560431.2
qCAT-R	CGGCTTCGTTCAGCACCTC	
qGR-F	CTTTGAACTTGAATCTTGCCCTAA	JX854448.1
qGR-R	CCCAGAGCAGACAGTCCACC	
qSOD-F	TGTGGACAAAATGCTGACCCTG	GU901214.1
qSOD-R	TTCCTCATTGCCTCCCTTCC	
qβ-actin-F	TTGCCGCACTGGTTGTTG	M25013.1
qβ-actin-R	TCCGTTTCTCACCTGATGTCTG	

### Statistical analysis

Data were analyzed using one-way ANOVA in SPSS 27.0. Statistical significance was set at *P* < 0.05 (^*^ or #) and *P* < 0.01 (^**^ or ##). Graphs were generated using GraphPad Prism 8.0 (GraphPad Software Inc., San Diego, CA, USA).

## Results

### Baicalein's anti-AH efficacy *in vitro*

**Figure 2:**
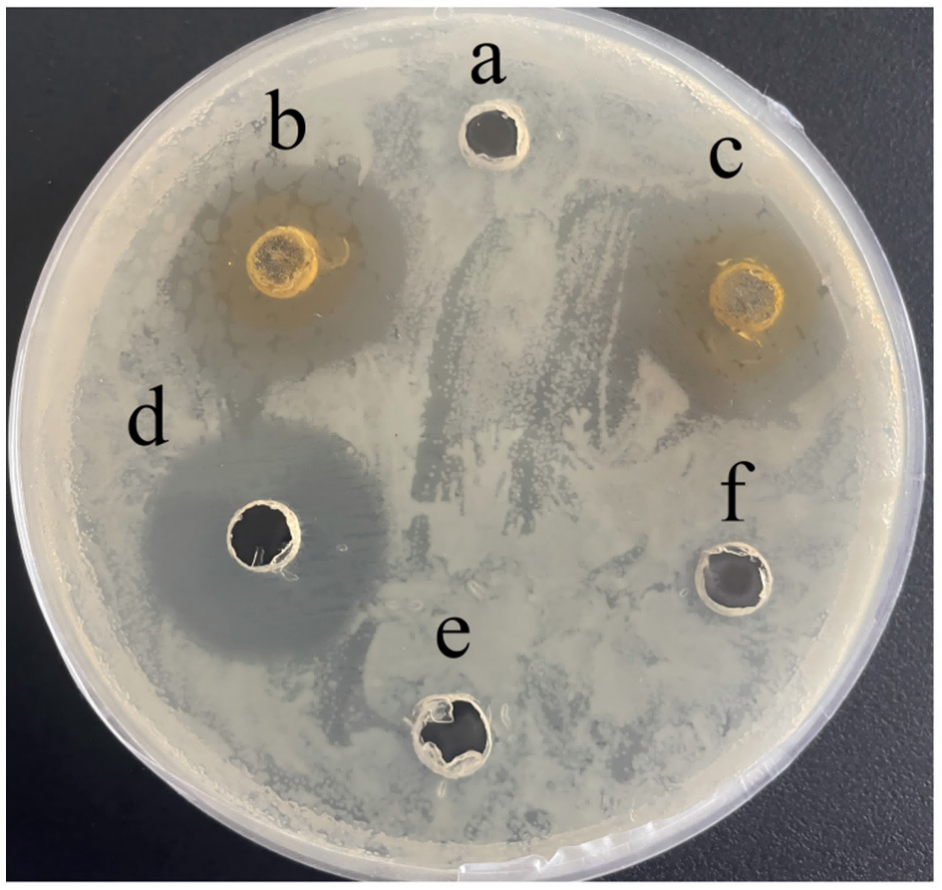
Inhibition zone of baicalein against AH *in vitro*. **(a)**, DMSO; **(b)**, 5 mg/mL baicalein; **(c)**, 10 mg/mL baicalein; **(d)**, 10 mg/mL kanamycin; **(e)**, PBS; **(f)**, H_2_O.

Baicalein exhibited significant antibacterial activity against *A. hydrophila* (AH). At concentrations of 5 mg/mL and 10 mg/mL, the inhibition zones measured 18.47 ± 0.40 mm and 20.08 ± 0.18 mm, respectively, compared to 23.26 ± 0.39 mm for 10 mg/mL kanamycin (positive control; Figure 2). The MIC and MBC values were determined to be 40 μg/mL and 80 μg/mL, respectively. Notably, at concentrations ≥80 μg/mL, no bacterial colonies were observed, demonstrating a potent bactericidal effect.

### Time-kill analysis

**Figure 3:**
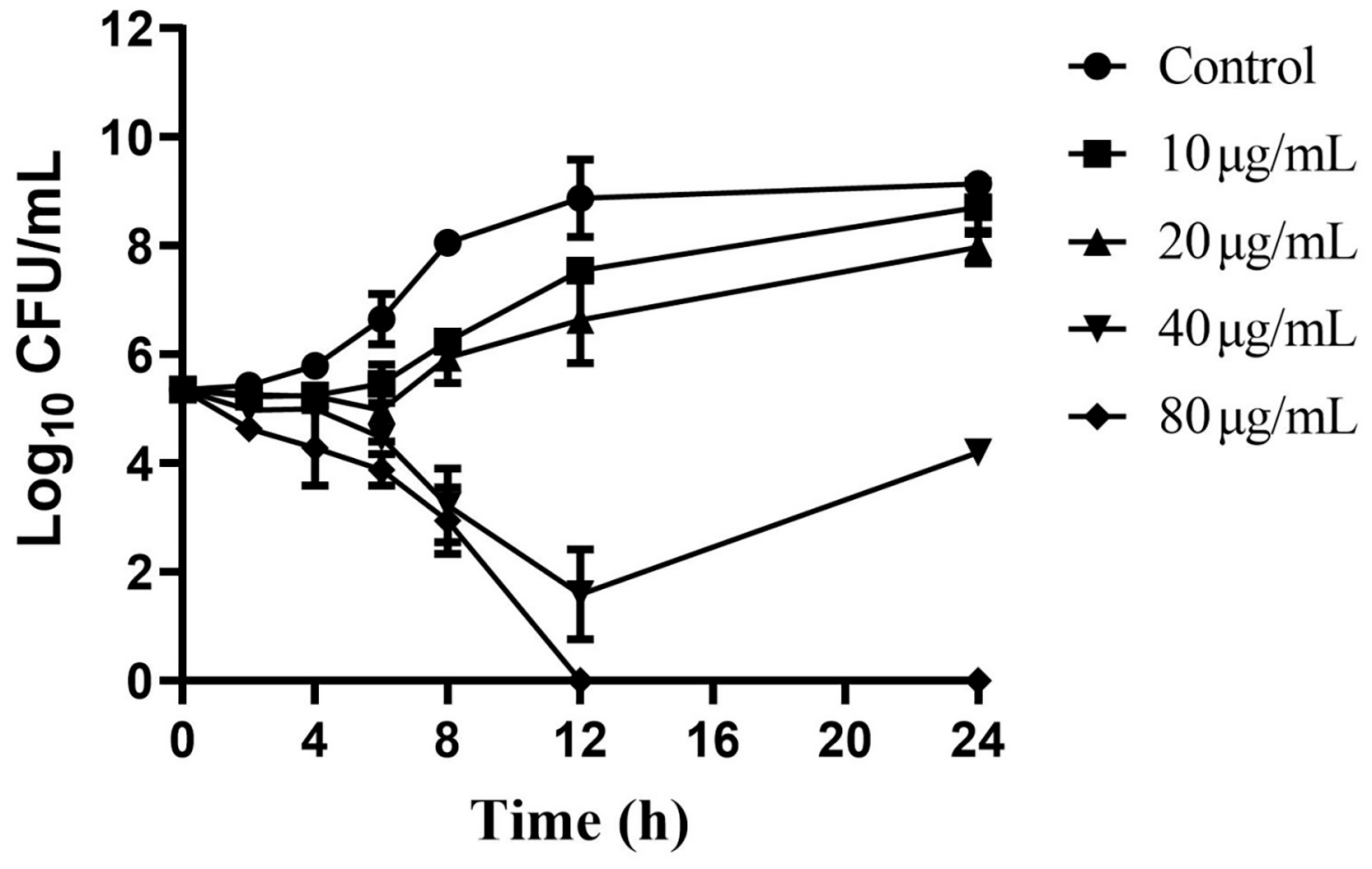
Time-kill curves of AH vs. different concentrations of baicalein. The baicalein concentrations were 10, 20, 40, and 80 μg/mL (as 1/4, 1/2, 1, and 2 MIC). Samples were taken at 0, 2, 4, 6, 8, 12, and 24 h to determine viable bacterial numbers.

Baicalein inhibited AH growth in a dose- and time-dependent manner. The time-kill curves (Figure 3) showed a substantial decrease in viable bacterial counts with increasing baicalein concentration. At 80 μg/mL (MBC), AH was completely eradicated within 12 h. At 40 μg/mL (1 × MIC), a significant reduction in bacterial counts was observed within the first 12 h, though some bacterial cells survived and proliferated by 24 h. These findings suggest that baicalein's antibacterial efficacy is dependent on both concentration and exposure time.

### Effects of baicalein on biofilm formation and motility

**Figure 4:**
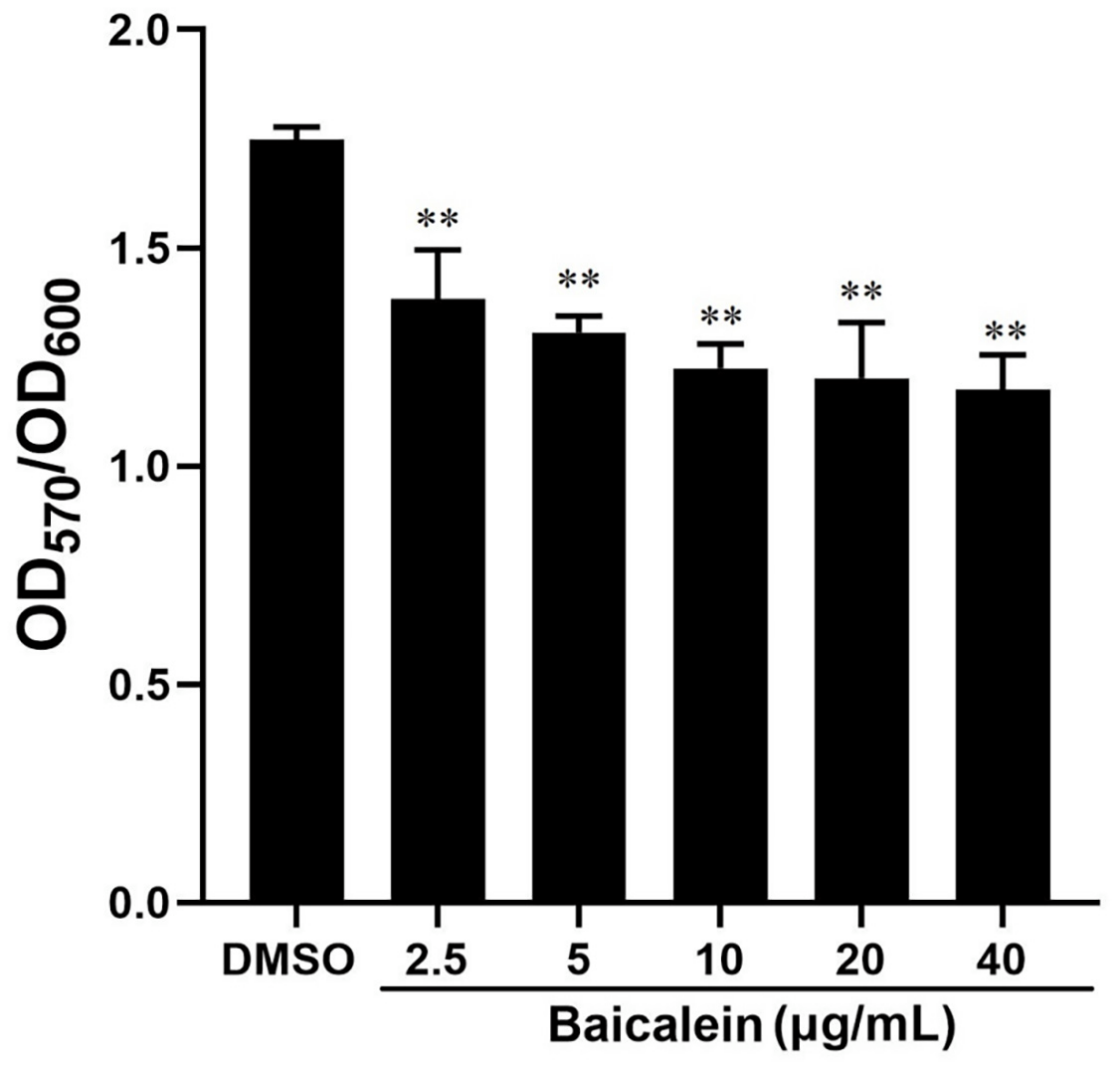
Effect of baicalein on the formation of AH biofilms. Different concentrations of baicalein (2.5, 5, 10, 20, and 40 μg/mL) were incubated with AH for 72 h. The biofilm formation was tested by the crystal violet staining method. **P < 0.01 compared to the DMSO treated group.

**Figure 5:**
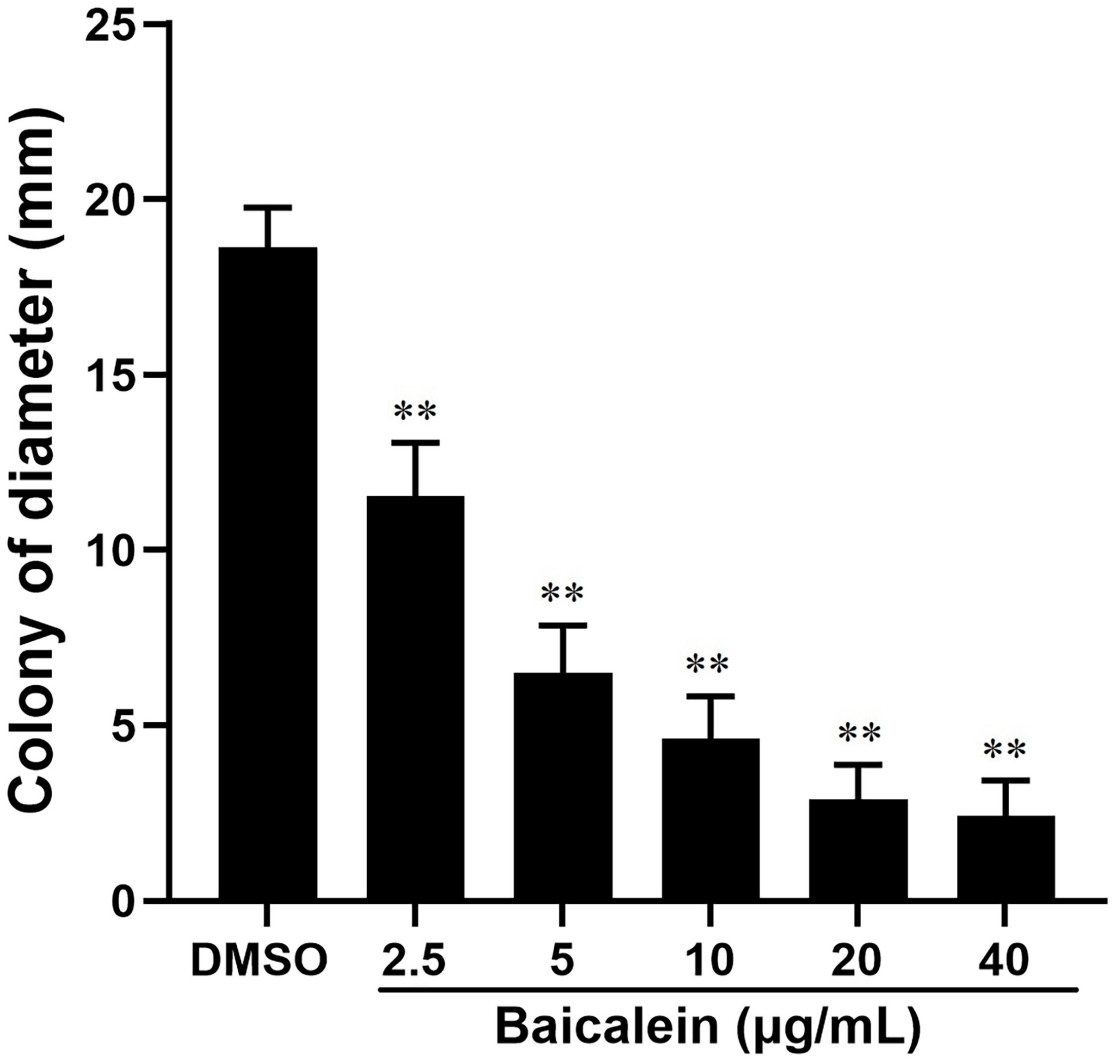
Effect of baicalein on the motility of AH. The assays were conducted on plates containing 0.3% agar in the presence of different concentrations of baicalein (2.5, 5, 10, 20, and 40 μg/mL) or DMSO as a control. The AH was incubated for 24 h. Colony diameters were measured using a caliper. **P < 0.01 compared to the DMSO treated group.

The crystal violet assay demonstrated that baicalein significantly reduced biofilm formation in a concentration-dependent manner, as indicated by a lower OD_570_ (relative value) in baicalein-treated groups compared to the DMSO-treated group (Figure 4). Biofilm inhibition ranged from 20.9% to 32.7% at concentrations between 2.5 and 40 μg/mL. Additionally, baicalein significantly impaired AH motility on semi-solid agar, as evidenced by reduced colony diameters in treated groups compared to controls (Figure 5).

### Baicalein disrupts the membrane structure of AH

**Figure 6:**
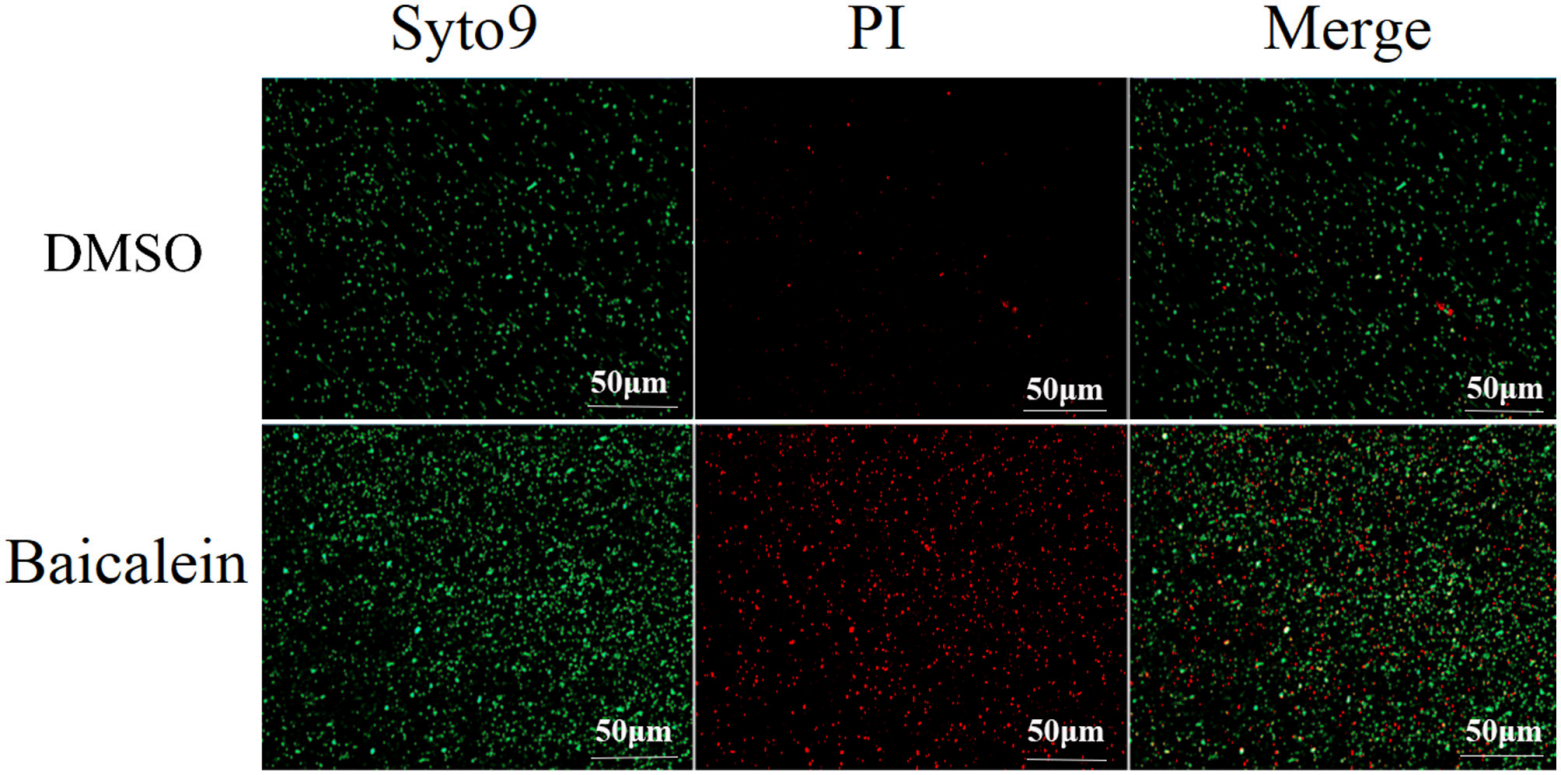
Fluorescence microscopy analysis of AH membrane permeability after treatment with baicalein. AH were incubated for 2 h with or without 2 MIC of baicalein and stained using a Live/Dead BacLight bacterial viability kit. Red and green immunofluorescence was observed under a fluorescence microscope.

**Figure 7:**
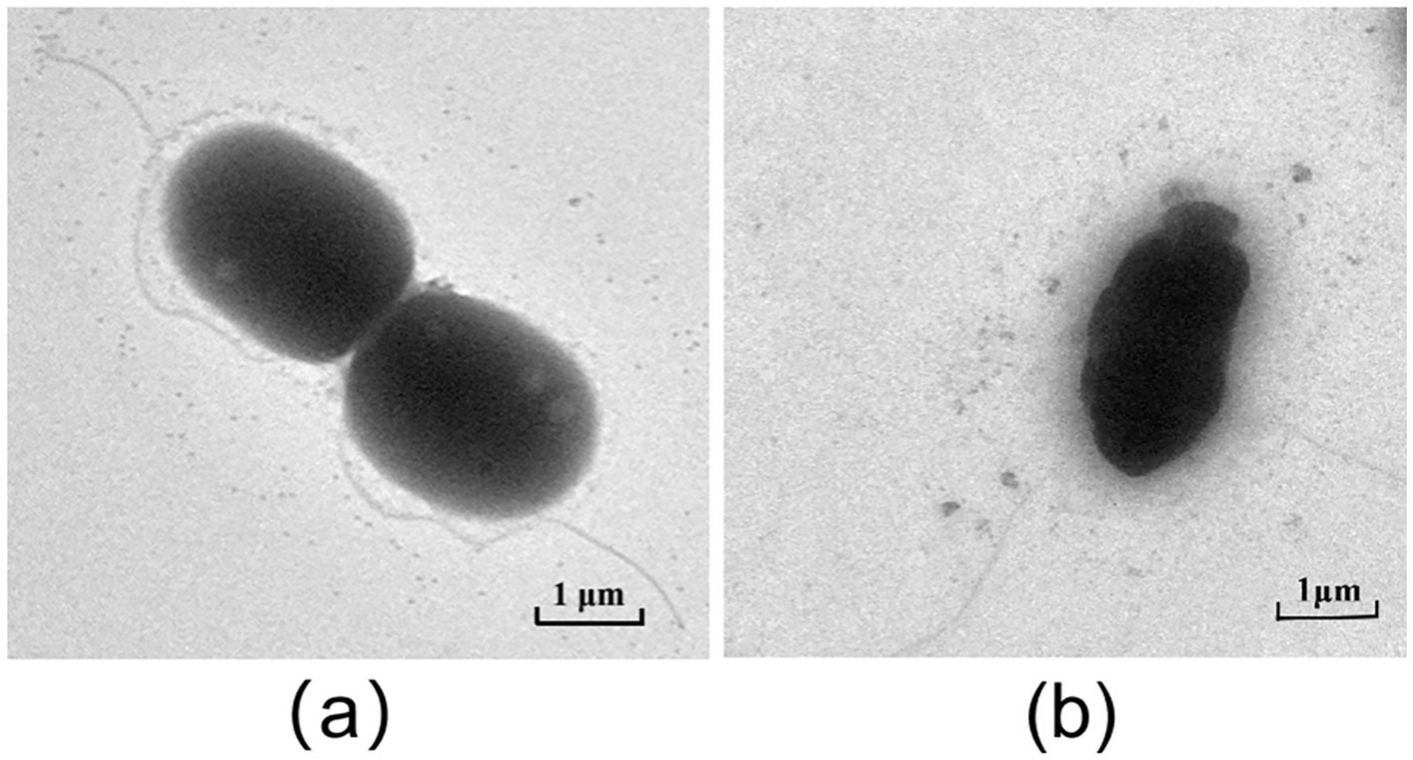
TEM results of AH after treatment with baicalein. **(a)**, DMSO treatment control; **(b)**, baicalein treatment group.

Live/Dead BacLight bacterial viability assays indicated that baicalein increased membrane permeability, as evidenced by enhanced red fluorescence in treated AH cells (Figure 6). Transmission electron microscopy (TEM) analysis further revealed that baicalein treatment caused significant structural damage, including membrane distortion and cell wall folding (Figure 7), whereas control cells maintained their structural integrity. These findings suggest that baicalein may inhibits AH growth by compromising membrane integrity and inducing cellular damage.

### *In vivo* antibacterial efficacy of baicalein

**Figure 8:**
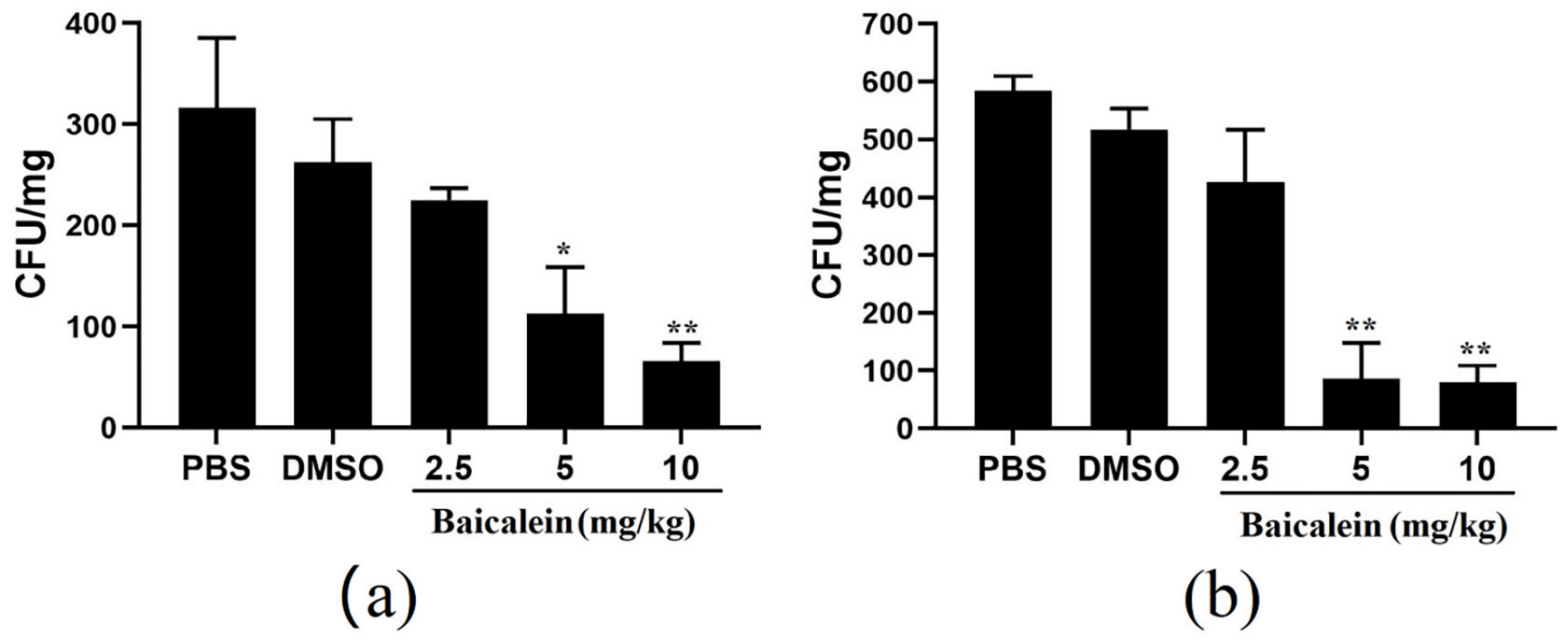
The number of colonies in the liver **(a)** and spleen **(b)** of grass carp after challenged with AH. *P < 0.05 and **P < 0.01 indicate significantly different from the DMSO group.

The antibacterial activity of baicalein was evaluated using an AH-infected grass carp model. Baicalein treatment significantly reduced bacterial counts in both liver and spleen tissues in a dose-dependent manner. In the liver, baicalein at 2.5, 5, and 10 mg/kg resulted in bacterial counts that were 3.9-, 2.3-, and 1.1 orders of magnitude lower than those in the DMSO -treated group, respectively (Figure 8a). In the spleen, bacterial counts were reduced by 6.5-, 6-, and 1.2 orders of magnitude lower at the corresponding baicalein doses (Figure 8b). These results demonstrate the potent *in vivo* bactericidal effect of baicalein against AH.

### Anti-inflammatory effects of baicalein in liver tissues

**Figure 9:**
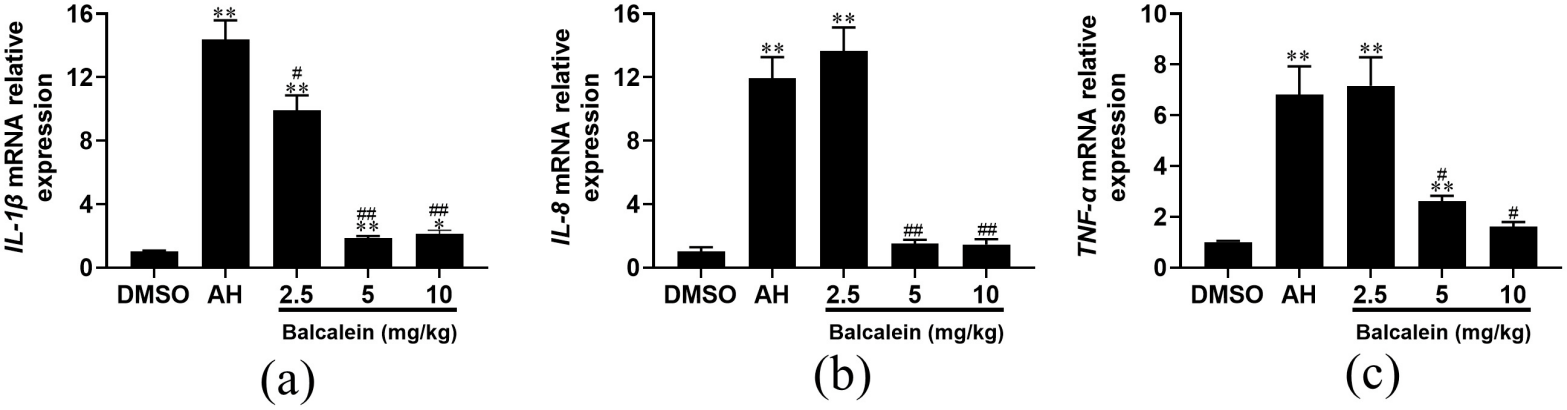
The mRNA expression levels of inflammatory genes after treatment with baicalein. The mRNA expression levels of *IL-1*β **(a)**, *IL-8* **(b)**, and *TNF-*α **(c)** were detected by qRT-PCR. Expression values are normalized against β-actin expression and data are expressed as the mean fold change from the control group or AH group. Three biological replicates were performed and the data are presented as mean ± SD (*n* = 3). *P < 0.05, **P < 0.01 indicate significantly different from the DMSO-treated group; ^#^*P* < 0.05 and ^##^*P* < 0.01 indicate significantly different from the AH group.

qRT-PCR analysis revealed that baicalein treatment led to a dose-dependent suppression of inflammatory cytokine expression (*IL-1*β*, IL-8*, and *TNF-*α) in liver tissues following AH infection. Compared to the AH-infected control group, *IL-1*β expression levels were 1.5-, 7.8-, and 6.8-fold lower in fish treated with 2.5, 5, and 10 mg/kg baicalein, respectively (Figure 9a). Similarly, *IL-8* expression levels were reduced by 0.88-, 7.9-, and 8.2-fold (Figure 9b), and *TNF-*α expression was decreased by 0.95-, 2.6-, and 4.2-fold, respectively (Figure 9c). These results suggest that baicalein effectively modulates AH-induced inflammatory responses in the liver.

### Upregulation of antioxidant-related genes by baicalein

**Figure 10:**
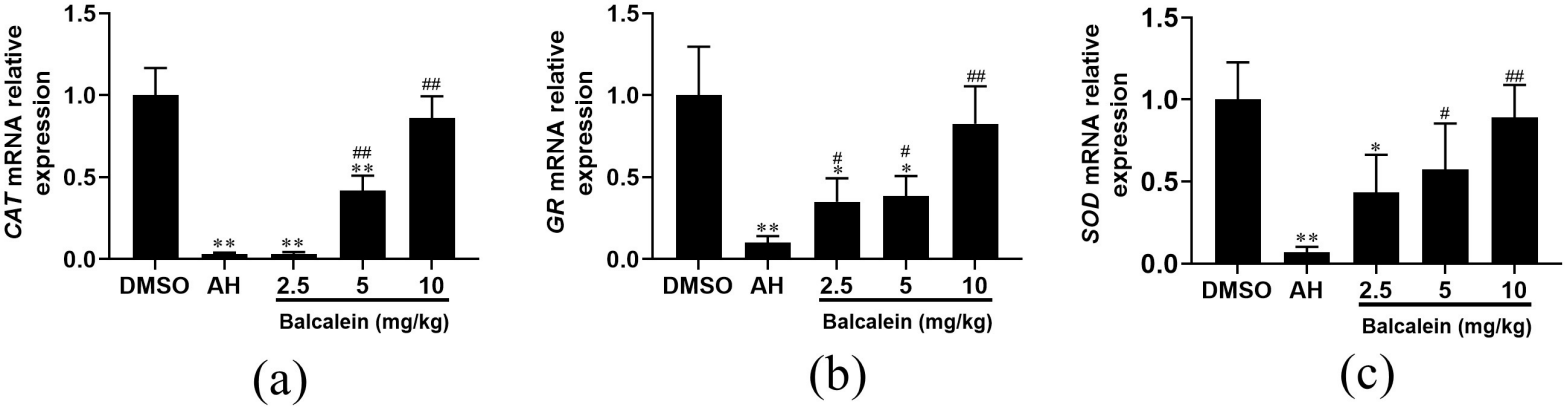
The expression of antioxidant-related genes after treatment with baicalein. The mRNA expression levels of *CAT* **(a)**, *GR* **(b)**, and *SOD* **(c)** were detected by qRT-PCR. Expression values are normalized against β-actin expression and data are expressed as the mean fold change from the control group or AH group. Three biological replicates were performed and the data are presented as mean ± SD (*n* = 3). *P < 0.05, **P < 0.01 indicate significantly different from the DMSO-treated group; ^#^*P* < 0.05 and ^##^*P* < 0.01 indicate significantly different from the AH group.

qRT-PCR analysis showed a significant increase in the mRNA expression of antioxidant-related genes (*CAT, GR*, and *SOD*) in baicalein-treated liver tissues compared to the AH-infected group. Specifically, *CAT* expression increased by 1.1-, 14.1-, and 29.1-fold in fish treated with 2.5, 5, and 10 mg/kg baicalein, respectively (Figure 10a). *GR* expression was elevated by 3.4-, 3.8-, and 8.1-fold (Figure 10b), and *SOD* expression increased by 6.4-, 8.4-, and 13.1-fold (Figure 10c). These findings indicate that baicalein enhances antioxidant responses in the liver, suggesting its potential role in oxidative stress regulation.

## Discussion

Baicalein, a major bioactive compound derived from *Scutellaria baicalensis*, has been widely studied for its pharmacological effects, particularly in infectious disease control. In our current study, baicalein exhibited significant antibacterial effects against AH, with MIC and MBC values of 40 μg/mL and 80 μg/mL, respectively. Previous studies reported MIC values of baicalein against *S. aureus* (64–256 μg/mL) (Chan et al., 2011; Chen et al., 2016) and *P. aeruginosa* (>1,024 μg/mL) (Luo et al., 2016). As some Chinese Herbal Medicine, the MIC and MBC of thymol to AH were 128μg/mL and 256μg/mL, respectively (Liang et al., 2022). The MIC of the water extract from gall, coptis, terminalia, rhubarb to AH were 7.81 mg/mL, 15.63mg/mL, 31.25 mg/mL, 62.50 mg/mL, respectively. The MBC were 15.63 mg/mL, 62.50 mg/mL, 62.50 mg/mL, 125.00 mg/mL, respectively (Su et al., 2012). The MIC values of antibiotics emodin and enrofloxacin against AH were found to be 100μg/mL and 9.375μg/mL respectively (Zhang et al., 2014). Baicalein demonstrated superior antibacterial activity against AH than other Chinese Herbal Medicine and closed to the antibiotics. The time-kill assay further confirmed its bactericidal properties at 80 μg/mL. Biofilm formation is a major contributor to antibiotic resistance (Rather et al., 2021). Using the crystal violet assay, our findings revealed that baicalein significantly inhibited AH biofilm formation and bacterial motility in a concentration-dependent manner, similar to previous reports on *S. aureus* (Chen et al., 2016) and *Streptococcus mutans* (Vijayakumar et al., 2021). While the crystal violet assay provides a useful quantitative assessment of biofilm formation by measuring total biomass and surface adhesion, it does not offer detailed structural insights. Future studies incorporating confocal microscopy or scanning electron microscopy (SEM) would allow for more comprehensive visualization of biofilm architecture and extracellular matrix components. Given that bacterial motility plays a critical role in biofilm development (Dressaire et al., 2015; Khong et al., 2021), the inhibition of AH motility by baicalein suggests a potential mechanism for biofilm disruption. Furthermore, baicalein was found to increase membrane permeability and disrupt AH cell membranes, consistent with prior studies on *S. aureus* (Wang et al., 2023; Paul et al., 2024). Many studies show that baicalein is a safe, low toxicity and few adverse effects (Paul et al., 2024). Studies have shown that the safe dosage is 500mg/kg in rat (Li et al., 2014). We use the dose of 10 mg/kg baicalein in the grass carp. The fish remains healthy with no adverse symptoms. *In vivo*, baicalein significantly reduced AH burden in grass carp, aligning with its reported efficacy against *H. pylori* (Chen et al., 2018) and *S. suis* infections in mice (Lu et al., 2021). Therefore, our findings indicated that baicalein could potentially be used as an antibiotic adjuvant drug for preventing aquatic animal infectious diseases caused by antibiotic-resistant bacteria (Güran et al., 2023; Liu et al., 2020; Wang et al., 2023). However, baicalein uses in aquaculture is limited by its poor water solubility, low bioavailability, and rapid breakdown in fish. It also degrades in alkaline water and is poorly absorbed when given orally. Direct water application is ineffective due to quick dilution. To improve its effectiveness, advanced delivery methods like nanoencapsulation are being explored to enhance stability, absorption, and targeting (Wang et al., 2024).

Microbial infections are regulated by host inflammatory responses, primarily driven by innate immunity. In aquaculture, *A. hydrophila* (AH)-induced sepsis poses a significant threat, as it triggers a systemic inflammatory response that can lead to severe tissue damage (Semwal et al., 2023). Pro-inflammatory cytokines play a crucial role in immune signaling cascades, with *IL-1*β*, IL-8*, and *TNF-*α being key mediators of inflammation (Megha et al., 2021). The liver is a central organ involved in immune regulation and systemic inflammation in fish (Wu et al., 2016). Moreover, previous studies have shown that baicalein exerts liver-protective effects (Yang et al., 2021), making it a relevant tissue for evaluating its anti-inflammatory potential. In this study, we analyzed inflammation levels in fish and found that AH infection significantly upregulated the expression of *IL-1*β*, IL-8*, and *TNF-*α. However, baicalein treatment markedly suppressed the AH-induced release of these cytokines in the liver. Previous research has demonstrated that baicalein reduces bacterial burden by inhibiting the expression of *IL-1*β*, IL-8, TNF-*α, and other inflammatory mediators, thereby mitigating excessive inflammatory responses in severe abdominal sepsis (Chen et al., 2022; Guo et al., 2019; Wang et al., 2023). These findings suggest that baicalein may exert a protective effect against AH-induced damage by attenuating inflammation.

Aquatic organisms produce various antioxidant enzymes and compounds in response to environmental stressors to counteract oxidative damage caused by reactive oxygen species (ROS) and other oxidants (Yang et al., 2022). Maintaining redox homeostasis is a critical component of the defense mechanisms that organisms have evolved to mitigate ROS-induced oxidative damage (Jiang et al., 2024). CAT and SOD are enzymatic antioxidants that play essential roles in neutralizing ROS (Rattanawong et al., 2021). Additionally, GR is involved in the glutathione redox cycle, facilitating the elimination of free radicals and peroxides while enhancing cellular resistance to oxidative stress (Chowdhury et al., 2020). Previous studies have shown that AH infection can induce excessive ROS production (Han et al., 2020), while baicalein exhibits antioxidative effects through its ROS-scavenging properties (Pan et al., 2021). In this study, we found that grass carp challenged with AH exhibited significantly reduced expression levels of *SOD, CAT*, and *GR* in liver tissues compared to the control group. However, baicalein treatment significantly upregulated the expression of these antioxidant-related genes, indicating its potential role in enhancing the antioxidant defense system in response to AH-induced oxidative stress.

It is important to note that while our study demonstrates transcriptional changes in inflammation- and antioxidant-related genes, it does not directly assess oxidative damage through biochemical markers such as malondialdehyde (MDA) (Rizzo, 2024). Future studies should incorporate such assays to more comprehensively evaluate the antioxidant efficacy of baicalein *in vivo*.

## Conclusions

Baicalein demonstrated potent antibacterial activity against AH *in vitro*, inhibiting bacterial motility, preventing biofilm formation, and increasing membrane permeability, ultimately leading to bacterial cell death due to cell structural damage. *In vivo*, baicalein effectively reduced AH burden, suppressed inflammatory responses, and enhanced antioxidant capacity in grass carp. These findings highlight baicalein's potential as a natural antimicrobial agent for controlling AH infections in aquaculture species.

## Data Availability

The datasets presented in this study can be found in online repositories. The names of the repository and accession numbers can be found in the Table 1.
